# Genomic and Chemical Investigation of Bioactive Secondary Metabolites From a Marine-Derived Fungus *Penicillium steckii* P2648

**DOI:** 10.3389/fmicb.2021.600991

**Published:** 2021-06-04

**Authors:** Guangshan Yao, Xiaofeng Chen, Huawei Zheng, Danhua Liao, Zhi Yu, Zonghua Wang, Jianming Chen

**Affiliations:** ^1^Institute of Oceanography, Minjiang University, Fuzhou, China; ^2^Fujian Universities Key Laboratory for Plant-Microbe Interaction, College of Life Science, Fujian Agriculture and Forestry University, Fuzhou, China

**Keywords:** isoquinoline alkaloids, anti-bacterial activities, coral-derived fungus, genome sequencing, *Penicillium steckii*

## Abstract

Marine fungi of the genus *Penicillium* are rich resources of secondary metabolites, showing a variety of biological activities. Our anti-bacterial screening revealed that the crude extract from a coral-derived fungus *Penicillium steckii* P2648 showed strong activity against some pathogenic bacteria. Genome sequencing and mining uncovered that there are 28 secondary metabolite gene clusters in P2648, potentially involved in the biosynthesis of antibacterial compounds. Chemical isolation and structural determination suggested citrinin is the dominant component of the crude extracts of P2648, and our further tests confirmed that citrinin showed excellent activities against various pathogenic bacteria. Moreover, the gene cluster containing a homolog of the polyketide synthase CitS was identified as the citrinin biosynthesis gene cluster through genetic analysis. Interestingly, three isoquinoline alkaloids were unexpectedly activated and isolated from the *Δcits* mutant and structural determination by using high-resolution electron spray ionization mass spectroscopy (HRESIMS), 1D, and 2D NMR. Further antibacterial assays displayed that compounds 1 and 2, but not compound 3, showed moderate activities against two antibiotic-resistant pathogenic bacteria with minimum inhibitory concentration (MIC) of 16–32 μg/ml. In conclusion, our results demonstrated that citrinin and isoquinoline alkaloids represent as the major antibacterial agents in the coral-associated fungus *P. steckii* P2648, and our genomic and chemical analyses present evidence in support of *P. steckii* P2648 as a potent natural products source for anti-bacterial drug discovery.

## Introduction

Bacterial resistance to antibiotics, such as extended-spectrum β-lactamase (ESBL)-producing *Escherichia coli*, has become a global public health issue (Lee et al., 2006). The global spread of bacterial resistance significantly increased patient mortality and morbidity due to the shortage of suitable antibiotics (Peirano and Pitout, 2019). Given the richness and diversity in chemistry and biological activity, the fungal secondary metabolites are regarded as inexhaustible sources for new antibacterial drugs (Abouelhassan et al., 2019). Species in the genus *Penicillium*, which are world widely distributed and mostly saprophytic, have since been considered as valuable resources of life-saving medicine and agrochemicals (Nielsen et al., 2017). The genus *Penicillium* consists of more than 350 of species, however, only a very limited number of them have been exploited for bioactive secondary metabolites. Among them, *Penicillium rubens* (also known as *Penicillium chrysogenum*) is one of the most prominent species and famous for its outstanding secondary metabolite penicillin, a paramount antibiotic for the therapy of infectious disease since World War II (Penalva et al., 1998). In addition, some other *Penicillium* fungi had been developed to produce series of pharmaceutical compounds, including the cholesterol-lowering drugs statins from *Penicillium citrinin* (Manu and Rogozea, 2016), mycophenolic acid from *Penicillium brevicompactum* (Anand and Srivastava, 2020), anti-cancer auranthine from *Penicillium aurantiogriseum* (Kalinina et al., 2018), and anti-Inflammation (3R, 7R)-7-acetoxyl-9-oxo-de-o-methyllasiodiplodin produced by *Penicillium* sp. (Liu et al., 2019).

Isoquinoline alkaloids constitute the largest classes of secondary metabolites with more than 2,500 defined structures, mainly produced by higher plants (Sato et al., 2007). Since the early 19th century, their complex structures and significant biological activities have attracted considerable attentions from researchers worldwide. However, so far, only a limited number of isoquinoline compounds were isolated from filamentous fungi, such as fumisoquins from *Aspergillus fumigatus* (Baccile et al., 2016), spathullins A and B from *Penicillium spathulatum* Em19 (Nord et al., 2019), imizoquins from *Aspergillus flavus* (Khalid et al., 2018), chaetolines A and B from *Chaetomium* sp (Ancheeva et al., 2018), and benzoisoquinoline-9-one from *Peyronellaea* sp. FT431(Li et al., 2019), and most were identified with anti-bacterial activities.

In our study, the genome of a coral-derived fungus *Penicillium steckii* P2648 was revealed by high-throughput sequencing, and founded to encode 28 of secondary metabolite gene clusters. The mycotoxin citrinin was identified as the major secondary metabolite of P2648 and showed antibacterial activities. The coding gene of citrinin cluster (*cits*) was genetically disrupted from P2648 genome. Besides, the disruption of *cits* compromised citrinin biosynthesis but caused the unanticipated accumulations of two anti-bacterial isoquinoline alkaloid compounds (Compound 1 and 2). Their chemical structures were classified by combining use of 1D, 2D-NMR, and MS. Taken together, our study demonstrated that citrinin endows the coral-derived *P. steckii* P2648 with prominent anti-bacterial features, while blocking the biosynthesis of citrinin will generate another two novel anti-bacterial isoquinoline alkaloids. Moreover, genetic dereplication approach will shed novel light on the study of secondary metabolites in marine-derived fungi.

## Materials and Methods

### Fungal Identification

The fungal strain was isolated from a gorgonian which was collected from the South China Sea. For growth and conidiation, the fungal strain was cultured on the PDA agar (potato juice 20%, dextrose 2%, and agar 1%), YES (sucrose 15%, yeast extract 2%, and agar 1%), or GMM (sucrose 2%, NaNO_3_ 3%, KH_2_PO_4_ 1%, KCl 0.5%, MgSO_4_ 0.1%, FeSO_4_ 0.01%, and agar 1%) for 7 days at room temperature in the dark. For molecular identification of fungal strain, the genomic DNA of fungal strain was extracted from approximately 100 mg of mycelium, scraped from PDA plates, using a thermolysis method as previously reported (Zhang et al., 2010). The quality and quantity of DNA samples were measured with the NanoDrop Spectrophotometer (Thermo Scientific, Wilmington, Germany). The ITS fragment was amplified using the primer pair *ITS1/ITS4* (Supplementary Table S2), and 18S rRNA gene was obtained from our sequencing data. A phylogenetic tree based on ITS and 18 s rDNA sequences was constructed by using the MAGA X with the neighbor-joining method (Kumar et al., 2018).

### Genome Sequencing

Genome sequencing of *P. steckii* P2648 was performed using the second-generation Illumina seq 4000 platform at Majorbio, China. The sequencing libraries were constructed using Illumina Paired-End DNA sample Prep Kit. Reads were assembled from raw data by using SOAPdenovo v2.04 program with a range of Kmers (17–92). Gene predictions were performed by using Maker2 program. Gene model annotations were carried out through sequence alignment in Cluster of Orthologous Groups of proteins (COG) database. Prediction of putative secondary metabolite biosynthesis gene cluster was carried out using the online AntiSMASH software. To use single-copy genes to plot species tree, the protein-coding genes from *P. steckii*, *Penicillium oxalicum*, *P. rubens*, *Penicillium griseofulvum*, *Penicillium brasilianum*, *Penicillium expansum*, and *Aspergillus nidulans* were downloaded from NCBI NR database. The single-copy genes were obtained by using OrthoFinder software. The phylogeny tree was constructed inferred from the amino acid sequences of 5,884 single-copy genes by IQ-TREE based on maximum likelihood algorithm. The phylogenetic tree was displayed by using iTOL.

### Gene Deletion

Gene deletion strategy in P2648 was slightly modified from that we previously established in *P. oxalicum* (Yao et al., 2015). Double-joint PCR was used to construct a gene deletion cassette, using the *hph* gene amplified from plasmid pSilent-1 as the selectable marker. All primers that were used to amplify the 5'- and 3'-flanks were listed in Supplementary Table S2 with the P2648 gDNA as the template. The entire gene deletion cassette was amplified with specific primers, using the 5'- and 3'-flanks for gene *citS* and *hph* mix as a template. The PCR products were transformed into the protoplasts of P2648 wild strain. For the preparation of protoplasts, approximately 10^9^ cfu/ml of conidia was inoculated into 100 ml PDB Broth, shaken at 150 rpm for 12 h at 30°C. Mycelia were collected by using four-layers of cotton gauzes, transferred to a 50 ml tube, and resuspended in 20 ml lysis filter-sterilized solution that contained 12 mg/ml lysing enzymes (Sigma, St Louis, MO, United States), 50 mM KC1, 1 M NaCl, and 1 mM CaCl_2_. After shaken at 60 rpm, 30°C for 3–4 h, protoplasts were harvested by filtering through a two-layer of lens paper. The obtained protoplasts were washed twice with a solution of 0.5 M KC1, 50 mM CaC1_2_, and 10 mmol Tris-HCl (pH 7.5). Fungal transformation and regeneration procedures were performed as described previously (Yao et al., 2015). Gene deletion mutants were preliminarily identified by PCR and then confirmed *via* RT-PCR. The primers used for verifying gene deletion mutants were listed in Supplementary Table S2.

### Fermentation, Extraction, Isolation, and Structure Determination

The fungus *Δcits* mutant was cultivated in the rice medium (80 g of rice, 3 g of natural sea salt, and 100 ml of H_2_O) in 1 L Erlenmeyer flask (20 flasks) at room temperature for 4 weeks. The fermented rice substrates were extracted three times with EtOAc (300 ml every time for each flask) and two times with MeOH (300 ml for each flask). The organic extracts were combined and concentrated under vacuum to afford a total extract (5.0 g), which was subjected to silica gel column chromatography (CC) using a step gradient elution with EtOAc-petroleum ether (0–100%) and then with MeOH-EtOAc (0–50%) to yield four fractions (Fr.1–Fr.4). Fr.3 was further subjected to silica gel CC, and eluted with CHCl_2_-MeOH (40:1, v/v) to obtain subfractions Fr.3-1–Fr.3-4, Fr.3-2 was further purified by semi-preparative high performance liquid chromatography (HPLC) eluted with MeOH-H_2_O (40:60, v/v) to give compounds 1 (14.0 mg) and 2 (19.0 mg). Fr.3-3 purified by Sephadex LH-20 CC with mixtures of CHCl_2_-MeOH (1:1, v/v) to yield compound 3 (12.0 mg).

Optical rotations were recorded on a JASCO P-1020 digital polarimeter. IR was acquired on a Nicolet-Nexus-470 spectrometer with of KBr Pellet Method. The 1D and 2D NMR spectra were recorded in MeOD on a JEOL JEM-ECP NMR spectrometer with tetramethyl silane (TMS) as the internal standard. All NMR assignments were based on the ^1^H-^1^H COSY, HSQC, and heteronuclear multiple bond correlation (HMBC) spectroscopic data. High-resolution electron spray ionization mass spectroscopy (HRESIMS) data were acquired on a Thermo MAT95XP high-resolution mass spectrometer (Thermo Fisher Scientific, United States). HPLC analysis and purification were performed on a Hitachi L-2000 HPLC system coupled with a Hitachi L-2455 photodiode array detector and using an analytical Kromasil column (250 mm × 4.6 mm, 5 mm) and a semi-prepared C18 column (250 mm × 10 mm, 5 mm), respectively. The mobile phase consisted of 0.1% formic acid in acetonitrile (A) and 0.1% formic acid in water (B) with a flow rate of 1 ml/min with 10 ml injection volume, and recorded at 254 nm.

### Antibacterial Activities Tests

The tested pathogenic bacterial strains include three common zoonotic pathogens (*E. coli* 25922, *Staphylococcus aureus* ATCC 2592, and *Pseudomonas aeruginosa* FRD1), two antibiotic-resistant bacteria [ESBL-producing *E. coli* (ATCC 35218) and vancomycin-resistant *Enterococcus faecalis* ATCC 51299]. The minimum inhibitory concentration (MIC) was determined by using a broth microdilution method according to the standards and guidelines recommended by Clinical and Laboratory Standards Institute (2012). Penicillin G sodium salt was used as anti-bacterial drug positive control.

## Results and Discussion

### Genome Sequencing

During our screening of the marine-derived fungi for producing anti-bacterial natural products, the crude extract of gorgonian-derived fungus P2648 showed strong inhibition on the growth of both Gram-negative *E. coli* and Gram-positive *S. aureus* (MIC 50–100 μg/ml). Based on morphological characteristics and phylogenetic analysis of 18S rRNA and ITS sequence, P2648 was classified into the genus *Penicillium* with the highest sequence similarity (99.8%) to *P. steckii* (Supplementary Figure S1). Until now, no genome of marine-derived *P. steckii* had been sequenced. To investigate comprehensively the potential of marine-derived fungus P2648 in production of secondary metabolites, genomic DNA was isolated from the mycelia of strain P2648 and sequenced by using the Illumina Hiseq 4000 sequencing platform. The obtained genome of P2648 was assembled into 961 scaffolds of about 33.4 megabases (Mb), which is larger than that of model *P. steckii* strain IBT 24891 (Table 1). And, compared to other fungi in *Penicillium* genus, P2648 has a relatively larger genome in size (Table 1). A total of 12,098 protein-encoding genes were predicted, which comparable to both soil-derived *P. steckii* and *Penicillium* fungi. Among them, 10,343 (85.49%) genes have functional annotations in the COG database (Supplementary Table S1). Referring to the functional classification of COG, and among them, 279 genes (2.7%) are involved into secondary metabolite biosynthesis, transport, or metabolism (Supplementary Table S1).

**Table 1:** Genome features of marine fungus P2648 and six *Penicillium* fungi.

Strains	Genome size (Mbp)	GC%	Proteins
P2648	33.4	46.4	10,343
*Penicillium steckii*	32.1	45.1	10,362
*P. citrinum*	31.5	46.2	9,754
*P. griseofulvum*	29.1	47.3	9,630
*P. rubens*	32.5	48.9	11,460
*P. expansum*	32.4	47.5	11,060
*P. oxalicum*	30.2	50.6	9,979

**Figure 1:**
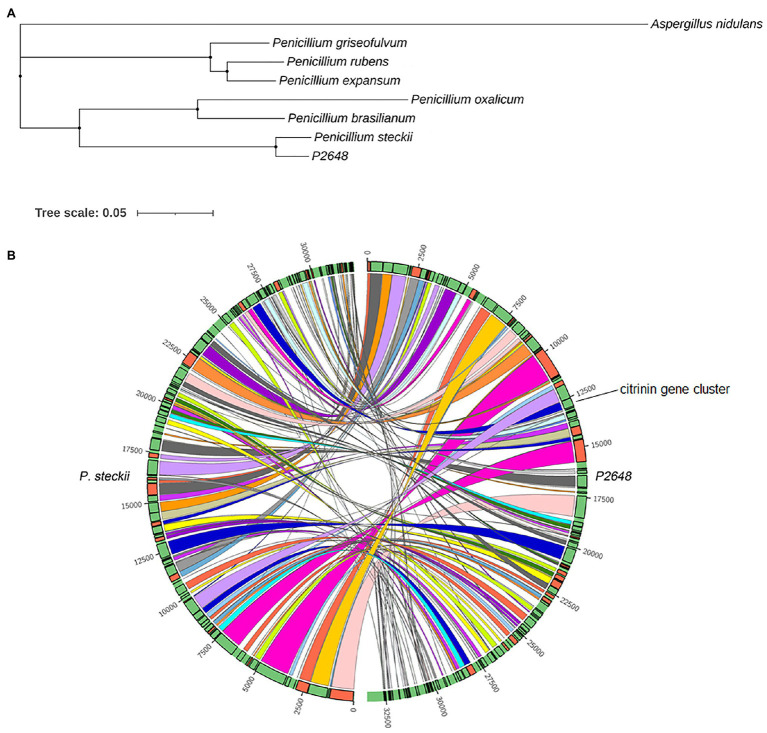
Phylogenetic and synteny analysis of P2648 with other fungal species. **(A)** The maximum likelihood phylogeny was constructed inferred from the amino acid sequences of 5,884 single-copy genes by IQ-TREE. The phylogenetic tree was displayed by using iTOL. The single-copy genes were obtained by OrthoFinder tool. *Aspergillus nidulans* from *Aspergillus* genus was used as the outgroup; **(B)** Sequence-synteny analyses between marine-derived P2648 and soil-species, and the Circo plots represent syntenic blocks between P2648 and *Penicillium steckii*.

To better understand the evolutional relationship, phylogenetic analysis was performed by using whole-genome alignment between P2648 and its relatives. The result showed that six *Penicillium* species and P2648 formed a clade with a high bootstrap support, in which P2648 showed closest relationship with *P. steckii* (Figure 1A). Therefore, together multi-loci DNA sequence and whole-genome alignment phylogenetic analysis confirmed that P2648 was one of *P. steckii* species. Sequence-synteny analyses between *P. steckii* and P2648 revealed that most of scaffolds showed synteny, however, extensive genetic divergences existed in the two *P. steckii* strains that were from different geographical regions (Figure 1B).

### Secondary Metabolite Gene Cluster

The secondary metabolites of filamentous fungi constitute a rich resource of bioactive natural products with antibiotic activities, such as penicillin from *P. chrysogenum*. Interestingly, genes encoding biosynthetic pathway for secondary metabolite are often located in a gene cluster on chromosome to form the secondary metabolite biosynthetic gene clusters (SMBGC; Keller, 2015). Mining P2684 genome for gene clusters involved in the biosynthesis of secondary metabolites was performed by using the online antiSMASH tool. A total of 28 (SMBGCs) were identified from the genome of P2648, including nine of polyketide (PKS), 10 of non-ribosomal peptide (NRPS)-like (NRPS-like), four of NRPS, two of terpene (TE), three of PKS-NRPS hybrid, and one betalactone (Table 2).

**Table 2:** Secondary metabolite gene cluster in P2648.

Cluster	Type	Similarity to known BGC
Cluster 1	NRPS-like	
Cluster 2	NRPS-like	
Cluster 3	T1PKS	100% Naphthopyrone
Cluster 4	NRPS-like	
Cluster 5	Betalactone	
Cluster 6	NRPS	100% Nidulanin A
Cluster 7	NRPS	
Cluster 8	NRPS	
Cluster 9	T1PKS	
Cluster 10	T1PKS	100% Sorbicillin
Cluster 11	T1PKS	
Cluster 12	NRPS-like	
Cluster 13	T1PKS	
Cluster 14	T1PKS	
Cluster 15	NRPS-like	
Cluster 16	NRPS,T1PKS	
Cluster 17	NRPS-like	
Cluster 18	NRPS-like	
Cluster 19	NRPS-like	
Cluster 20	NRPS-like,T1PKS	
Cluster 21	NRPS-like	
Cluster 22	Terpene	50% Squalestatin S1
Cluster 23	NRPS-like	
Cluster 24	NRPS,T1PKS	
Cluster 25	T1PKS	
Cluster 26	T1PKS	50% TAN-1612
Cluster 27	Terpene	
Cluster 28	T1PKS	

**Figure 2:**
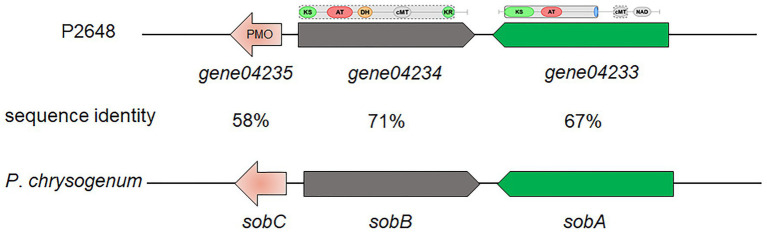
The putative sorbocillin biosynthetic gene cluster of P2648 and the comparison of this cluster with the sorbocillin cluster reported for *P. chrysogenum*. PMO, monooxygenase; KS, ketosynthase domain; AT, acyltransferase; DH, dehydratase domain; cMT, carbon methyltransferase; KR, ketoreductase domain; and NAD, male sterility protein.

Based on antiSMASH prediction, we found that *P. steckii* P2648 has the potential to produce sorbocillin (Table 2 and Figure 2). Sorbocillin and its derivatives, sorbicillinoids are a class of biologically active and structurally diverse fungal polyketides (Salo et al., 2016). Cluster 10 is consisted of three genes, two polyketide synthase (gene04233 and gene04234), and one FAD-dependent monooxygenase (gene04235). The two-polyketide synthases 04233 and 04234 showed high sequence similarities with the highly reducing iterative PKS SorA and the non-reducing iterative PKS SorbB, respectively (Figure 2). In sorbocillin biosynthesis, these two megaenzymes were reported to assemble the polyketide skeleton. And, the FAD-dependent monooxygenase 04235 from Cluster 10 showed high sequence similarity with the post-modification enzyme SobC (Figure 2). Together, our *in silico* analysis suggested that P2648 could produce sorbocillin and its analogs.

The polyketide cluster encoded by Cluster 3 showed 100% sequence similarity to the naphthopyrone cluster from *A. nidulans*, which responsible for biosynthesis of the yellow conidial wall pigment (Mayorga and Timberlake, 1992). In addition, Cluster 6 was predicted to biosynthesize secondary metabolites with structurally similar to nidulanin A and Cluster 22 showed high similarity to the terpene cluster squalestatin S1. Cluster 26 is composed of six genes, encoding FAD-dependent monooxygenase (gene10770), oxidoreductase (gene10771), o-methyltransferase (gene10772), monooxygenase (gene10773), non-reducing polyketide synthase (gene10774), and a transcription factor (gene10775). Among them, four of genes showed high sequence similarities with their counterparts involved in biosynthesis of TAN-1612 (Li et al., 2011). Therefore, we hypothesized that Cluster 26 potentially synthesize similar polyketide compounds.

Intriguingly, secondary metabolite products encoded by most of these SMBGCs in P2648 genome could not be assigned. In particular, three PKS/NRPS hybrid SMBGCs (Cluster 16, Cluster 20, and Cluster 24) were identified in P2648 genome, their secondary metabolite products remain to be determined. Also, we predicted 10 NRPS-like BGC gene clusters in P2648, no any compound could be assigned by bioinformatic analysis. Together, we have performed a comprehensive analysis and provide valued information on gene clusters for secondary metabolite biosynthesis by using both antiSMASH and gene cluster homology comparisons.

### Citrinin Is the Major Anti-bacterial Compound Produced by the Coral-Derived Fungus *P. steckii* P2648

To chemically identify the antibacterial agents, scaled fermentation of P2648 was performed by using a solid rice medium for 10 days. The ethyl acetate extracts of the fermentation were fractionated by using repeated positive and negative silica chromatography. A golden-yellow needle crystal (14 g) was obtained, which accounting for the largest population of total organic extracts (about 67%). Its structure was then identified to be as citrinin by comparing its ^1^H NMR and ^13^C NMR (Table 3) with previously reported NMR data (Blanc et al., 1995).

**Table 3:** ^1^H (400 MHz) and ^13^C (100 MHz) NMR spectroscopic data for citrinin (CDCl_3_).

Position	Citrinin	
	*δ*_C_, type	*δ*_H_, mult. *J* in Hz
1	162.9, CH	8.25, s
3	81.7, CH	4.80, dq. 6.6, 0.8
4	34.6, CH	3.00, dq. 7.2, 0.9
4a	139.2, C	
5	123.1, C	
6	183.8, C	
7	100.3, C	
8	177.2, C	
8a	107.4, C	
9	18.2, CH_3_	1.36, d. 6.7
10	18.5, CH_3_	1.24, d. 7.2
11	9.4, CH_3_	2.03, s
12	174.6, C	
8-OH		15.1, s
12-OH		15.9, s

### Identification of the Citrinin Biosynthetic Gene Cluster

**Figure 3:**
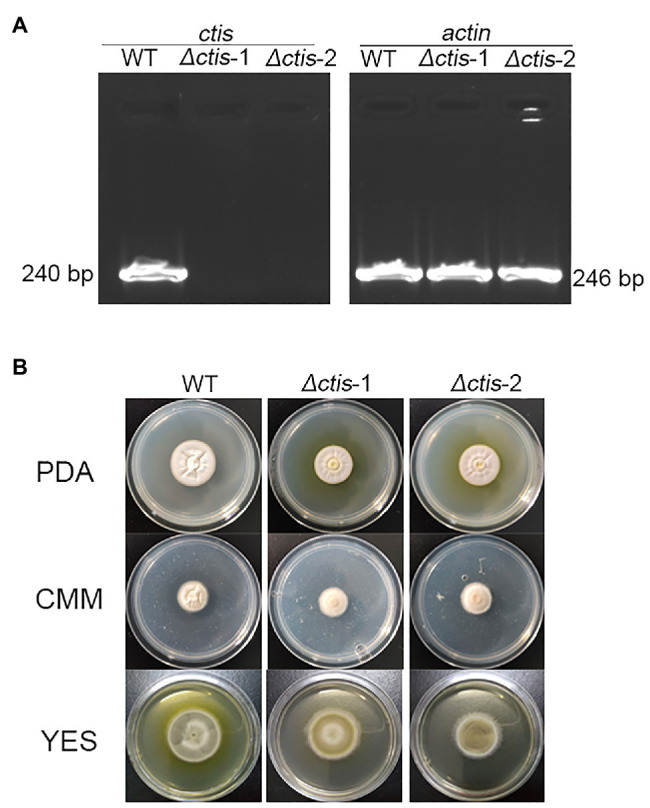
Deletion of *citS* gene in P2648. **(A)** RT-PCR verification of gene deletion, the transcripts of *actin* gene were used as the control; **(B)** Conidia of *Δcits* and wild-type (WT) were inoculated on the PDA, CMM, and YES plates, culturing at 30°C for 7 days.

**Figure 4:**
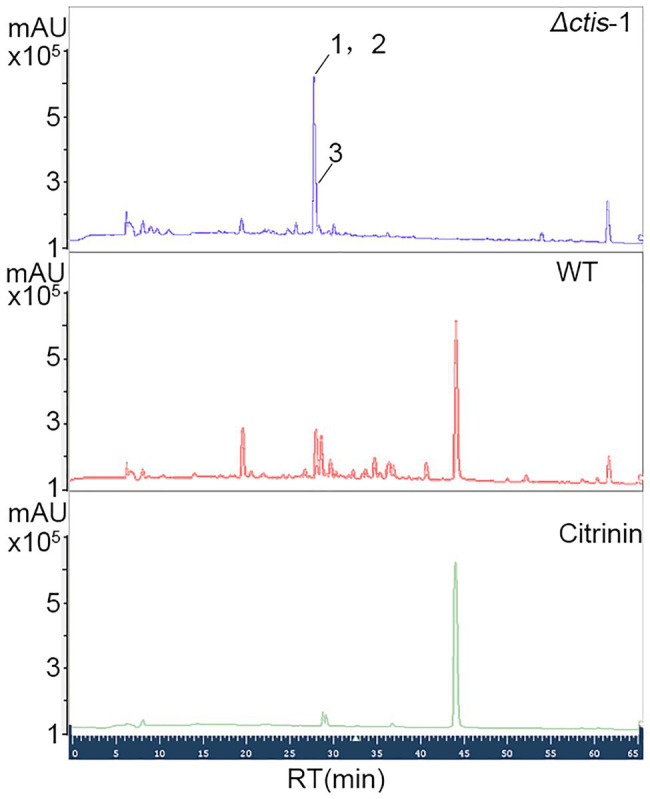
High performance liquid chromatography (HPLC) analysis of secondary metabolite profiles of both *Δcits* mutant and WT strains. Conidia of *Δcits* and WT were inoculated in the rice medium and cultured at room temperate for 21 days, the secondary metabolites were extracted by using EtOAc, and then analyzed by HPLC.

No gene cluster for citrinin biosynthesis could be ascertained by antiSMASH analysis (Table 2). Therefore, to identify the gene cluster for citrinin biosynthesis, a manual BLAST search by using CitS coding gene from *Monascus ruber* as a query against the *P. steckii* P2648 genome was performed, and results showed that a putative citrinin polyketide cluster containing nine genes were identified (Table 4). And, the entire citrinin gene cluster showed synteny between P2648 and *P. steckii*. The gene04630 (the homolog of *cits*) encodes the polyketide synthase, synthesizing triply methylated pentaketide skeleton by using methylmalonyl-SCoA as the building block (He and Cox, 2016). The methyl at 12-site of methylated pentaketide is progressively oxidized to carboxylic acid by non-heme iron oxidase CitB, FAD-dependent oxidoreductase CitC, and followed by CitD. Then, the 3-carbonyl is reduced to ether by the dehydrogenase CitE. CtnR and CtnC are the pathway-specific transcription factor and transporter, respectively. To confirm the function of the putative polyketide cluster in the citrinin biosynthesis, the open reading frame (ORF) region of PKS gene *cits* was deleted from *P. steckii* wild-type strain P2648 (WT) genome by replacing with hygromycin phosphate transferase (*hph*) gene. A total of 21 hygromycin-resistance transformations were obtained by three cycles of genetic transformation, and two deletion mutants were further verified through RT-PCR with gene-specific primers (Figure 3A). Absence of *cits* gene had no detectable defects on both vegetative growth and conidiation of P2648 on various media (Figure 3B). As anticipated, the *Δcits* mutant produced a significant difference in secondary metabolite profiles when compared with WT (Figure 4). Citrinin dominates the fermentation extracts of WT, but no citrinin can be detected in *Δcits*, suggesting that CitS is essential for citrinin biosynthesis. Besides, intriguingly, as shown in Figure 4, several peaks with retention time from 26 to 28 min were emerged and enhanced in *Δcits* when compared with WT.

**Table 4:** The citrinin biosynthesis gene cluster.

Gene ID	Description	Length	Functional annotation	Identity (%)
gene04631	CtnC	859	Transporter	79.33
gene04630	CitS	2,584	Polyketide synthase	74.81
gene04629	CitA	262	Oxydoreductase	78.76
gene04628	CitB	328	Iron/ascorbate oxidoreductase	81.76
gene04627	CtnR	552	Zn(II)_2_Cys_6_ transcriptional regulator	78.93
gene04626	CtiD	501	Aldehyde dehydrogenase	85.20
gene04625	unknown	128	Hypothetical protein	85.27
gene04624	CitE	915	Dehydrogenase	72.50
gene04623	CitC	463	FAD-dependent oxidoreductase	74.94

### Three Isoquinoline Alkaloids Were Unexpectedly Accumulated in the *Δcits* Mutant

**Figure 5:**
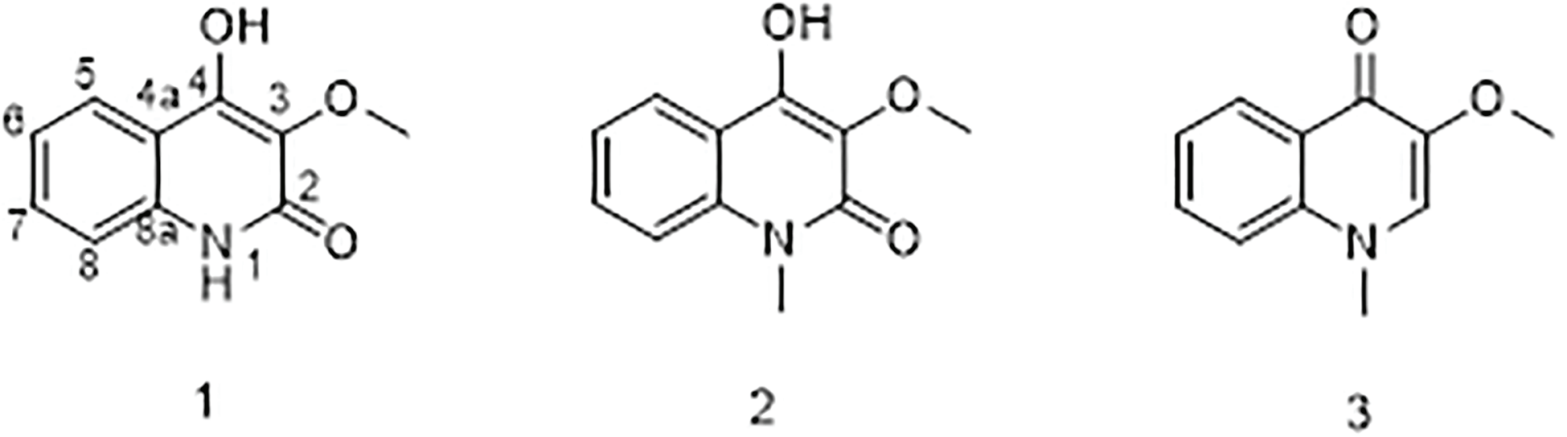
Structures of compounds 1–3.

To further mine the induced metabolite potentials by obstructing citrinin synthesis, the secondary metabolites of *Δcits* were harvested by scaled fermentation. The organic extracts were fractionated by repeated silica gel chromatography, Sephadex LH-20, and semi-preparative HPLC, and finally obtained compounds 1–3 (Figure 5).

**Figure 6:**
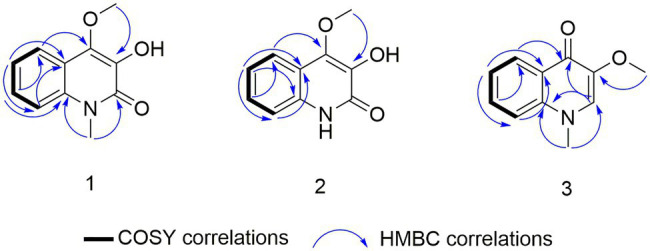
COSY and key heteronuclear multiple bond correlation (HMBC) correlations for 1–3.

Compound 1 was obtained as a white powder. Its molecular formula was determined to be C_10_H_9_NO_3_ on the basis of the HRESIMS (*m/z* 192.0653 [M + H]^+^; calcd for C_10_H_10_NO_3_, 192.0655) and the ^13^C NMR data. The ^1^H NMR data (Table 5) displayed four aromatic proton signals at *δ*_H_ 7.92 (1H, d, *J* = 8.0, 0.8 Hz, H-5),7.48 (1H, ddd, *J* = 7.2, 8.8, 1.2 Hz, H-7), 7.30 (1H, d, *J* = 8.0 Hz, H-8), and 7.24 (1H, ddd, *J* = 7.2, 8.0, 1.2 Hz, H-6); and an oxygenated singlet methyl proton resonance at *δ*_H_ 3.85. Additionally, the ^13^C and HSQC spectra showed 10 carbon signals, attributable to one ester group carbonyl resonating at *δ*_C_ 161.6, eight aromatic or olefinic carbon signals at *δ*_C_ 153.0, 135.3, 130.4, 129.5, 122.6, 121.9, 115.9, and 114.9, and one oxygenated methyl signal at *δ*_C_ 59.2. The aforementioned NMR data indicated the presence of one 1,2 disubstituted aromatic ring and one olefinic bond and these groups accounted for six out of the seven degrees of unsaturation, requiring one additional ring in 1. Analysis of the COSY spectrum showed correlations of H-5/H-6, H-6/H-7, and H-7/H-8 (Figure 6). Based on the HMBC cross-peaks from H-5 to C-4/C-7/C-8a (Figure 6), from H-6 to C-4a/C-5/C-8a/C-8, from H-7 to C-5/C-8/C-8a, as well as from H-8 to C-4/C-4a/C-6 were used to connect the aromatic ring with the olefinic bond. The HMBC correlations from oxygenated methyl proton *δ*_H_ 3.85 to C-3 (*δ*_C_ 130.4) demonstrated the groups of ▬OCH_3_ was anchored at C-3. Herein, compound 1 was elucidated as an isoquinoline alkaloid, 4-hydroxy-3-methoxy-2(1*H*)-quinolinone.

**Table 5:** ^1^H (400 MHz) and ^13^C (100 MHz) NMR spectroscopic data for 1 and 2 (methanol-*d*_4_).

Position	1		2	
	*δ*_C_, type	*δ*_H_, mult. *J* in Hz	*δ*_C_, type	*δ*_H_, mult. *J* in Hz
1-CH_3_			28.4, CH_3_	3.71, s
2	161.6, C		161.2, C	
3	130.4, C		130.2, C	
4	153.0, C		152.0, C	
3-OCH_3_	59.2, CH_3_	3.85, s	59.0, CH_3_	3.82, s
4a	115.9, C		116.8, C	
5	122.6, CH	7.92, d. 8.0, 0.8	123.1, CH	8.02, d. 7.6
6	121.9, CH	7.24, ddd. 8.0, 7.2, 1.2	121.9, CH	7.30, ddd. 8.0, 6.8, 0.8
7	129.5, CH	7.48, ddd. 8.8, 7.2, 1.6	129.9, CH	7.60, ddd. 8.8, 7.2, 1.6
8	114.9, CH	7.30, d. 8.0	114.1, CH	7.51, d. 7.6
8a	135.3, C		136.8, C	

Compound 2 was also obtained as a white powder with the molecular formula C_11_H_11_NO_3_ from HRESIMS (*m/z* 206.0808 [M + H]^+^; calcd for C_11_H_12_NO_3_, 206.0812), suggesting that 2 has one CH_2_ mass unit more than 1. A careful comparison of the ^1^H and ^13^C NMR spectra of 2 (Table 6) with those of 1 showed a close structural relationship, with the only difference being the oxygenated methyl proton signal at *δ*_H_ 3.71 and the oxygenated methyl signal at *δ*_C_ 28.4. According to the HMBC cross-peaks from ▬NCH_3_ (*δ*_H_ 3.71) to C-2 and C-8a, the oxygenated methyl group was attached to the amide group. Therefore, compound 2 was characterized as 4-hydroxy-3-methoxy-1-methyl-2(1*H*)-quinolinone.

**Table 6:** ^1^H (400 MHz) and ^13^C (100 MHz) NMR spectroscopic data for 3 (methanol-*d*_4_).

Position	3	
	*δ*_C_, type	*δ*_H_, mult. *J* in Hz
1-CH_3_	39.8, CH_3_	3.99, s
2	130.8, CH	7.99, s
3	142.9, C	
3-OCH_3_	56.8, CH_3_	3.89, s
4	171.6, C	
4a	125.3, C	
5	125.5, CH	8.40, d. 8.4
6	122.9, CH	7.43, ddd. 8.0, 6.4, 1.2
7	131.7, C	7.78, ddd. 8.4, 6.8, 1.6
8	115.8, CH	7.72, d. 8.40
8a	139.0, C	

Compound 3 with the molecular formula C_11_H_11_NO_2_ as determined by HRESIMS (*m/z* 190.0858 [M + H]^+^; calcd for C_11_H_12_NO_2_, 190.0863), was isolated as the white powder. The ^1^H NMR spectrum (Table 6) was very similar to that of 2, with the notable difference being the addition of a singlet proton resonance at *δ*_H_ 7.99 in 3. Comparison of the ^13^C NMR spectra of 3 with those of 2 displayed an unsubstituted olefinic carbon signal at *δ*_C_ 130.8. Combining the HMBC correlations from ▬NCH_3_ (*δ*_H_ 3.71) to C-2 and C-8a and from ▬OCH_3_ (*δ*_H_ 3.89) to C-3, the structure of compound 3 was assigned as 3-methoxy-1-methyl-4(1*H*)-quinolone.

The unexpected accumulation of three isoquinoline alkaloids after the deletion of *citS* might cause by, at least, the redistribution of biosynthetic precursors. More specifically, in WT strain, citrinin is the dominant secondary metabolite, which obstructs the biosynthesis of other compounds by consuming most of the universal precursor substances, such as acetyl-CoA and malonyl-CoA. Elimination of the citrinin biosynthesis might increase the reserve pools of these precursors, which would be redirected into the biosynthesis pathway of isoquinoline alkaloids. Additionally, the deletion of *citS* also resulted in a low SM background, which consequently contributed to the discovery of those isoquinoline alkaloids with formerly low synthetic levels.

### Biological Activity Assay

The antibacterial activities of three isoquinoline alkaloids and citrinin against a variety of pathogenic bacteria were assessed using the broth microdilution method to determine the MICs (Table 7).

**Table 7:** Antibacterial activity assays.

Compound	MIC (μg/ml)				
	*E. coli* ATCC 25922	*E. coli* ATCC 35218	*S. aureus* ATCC 2592	*E. faecalis* ATCC 51299	*P. aeruginosa* FRD1
1	32	32	16	32	<512
2	16	16	<512	16	<512
3	<512	<512	<512	<512	<512
Citrinin	16	16	16	4	<512
Penicillin sodium salt	4	256	8	8	4

Citrinin displayed excellent biological activities against all tested pathogenic bacteria with MIC of from 4 to 16 μg/ml, except for *P. aeruginosa* FRD1. Compound 1 exhibited an MIC of 16–32 μg/ml against all tested pathogens expect for *P. aeruginosa* FRD1, and compound 2 also showed an MIC of 16 μg/ml against three pathogenic bacteria, including two *E. coli* and one *E. faecalis*. The most noteworthy result is that both compounds 1 and 2 showed obvious activities against two drug-resistant microbial bacterial pathogens *E. coli* ATCC 35218 and *E. faecalis* ATCC 51299, implying a different action mechanism from common antibiotics. Compound 3 did not show detectable biological activities against any of tested pathogenic bacteria. Compared with compounds 1 and 2, hydroxy at position 4 was oxidized to a carbonyl in compound 3, suggesting that the 4-hydroxy is essential for the anti-bacterial activities of isoquinoline alkaloids.

## Conclusion

In the study, we reported the draft genome of a coral-derived fungus P2648. Whole-genome phylogenetic analysis demonstrated that P2648 is a novel strain of *P. steckii* species. The genome data and our bioinformatic analysis firstly revealed that *P. steckii* contains a large number of secondary metabolite cluster without assigned compounds. Further, our chemical investigation indicated that citrinin was identified as the dominant antibacterial agent of P2648, and a CitS-containing SMBGC was proved to be involved in citrinin biosynthesis in P2648. Surprisingly, three isoquinoline alkaloids were accumulated after blocking the biosynthesis of citrinin, and two of isoquinoline alkaloids showed moderate activities against two antibiotic-resistant pathogenic bacteria. In conclusion, our genomic and chemical analyses present evidence in support of *P. steckii* P2648 as a potent natural products source for anti-bacterial drug discovery.

## Data Availability Statement

The whole genome sequence of P. steckii P2648 has been deposited at NCBI database under the Bioproject number PRJNA671565.

## Author Contributions

GY, ZW, and JC conceived and designed the research work. GY, HZ, XC, DL, and ZY performed the experiments and analyzed the data. GY wrote the manuscript. HZ, XC, ZW, and JC revised the manuscript. All authors contributed to the article and approved the submitted version.

### Conflict of Interest

The reviewer LL declared a shared affiliation, with no collaboration, with three of the authors DL, ZY, and ZW to the handling editor at the time of the review.

The remaining authors declare that the research was conducted in the absence of any commercial or financial relationships that could be construed as a potential conflict of interest.

## Supplementary Material

Supplementary Figure S1 Phylogenetic tree analysis of P2648 and related filamentous fungi. Phylogenetic tree of P2648 and all related strains were reconstructed based on a 18S RNA gene alignment using the MEGA X software package with the neighbor-joining method. Bootstrap analysis (1,000 replications) was used to provide confidence estimates for phylogenetic tree topologies, and the percentage values are indicated at nodes.

Supplementary Table S1 Protein-encoding gene annotations.

Supplementary Table S2 Primers used in this study.
