# Supplementary figures and images for: Genomic and Chemical Investigation of Bioactive Secondary Metabolites From a Marine-Derived Fungus Penicillium steckii P2648
Source: Front Microbiol. 2021 Jun 4;12:600991. doi: 10.3389/fmicb.2021.600991 (PMC8211754; doi:10.3389/fmicb.2021.600991)

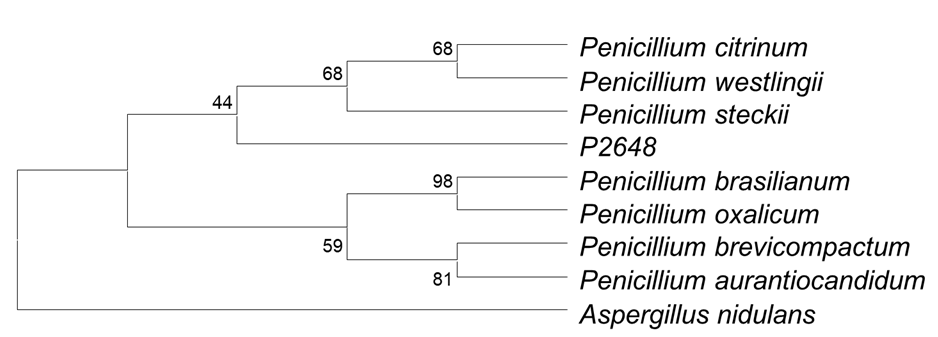

Supplement: Supplementary Figure S1 — Phylogenetic tree analysis of P2648 and related filamentous fungi. Phylogenetic tree of P2648 and all related strains were reconstructed based on a 18S RNA gene alignment using the MEGA X software package with the neighbor-joining method. Bootstrap analysis (1,000 replications) was used to provide confidence estimates for phylogenetic tree topologies, and the percentage values are indicated at nodes. [file Image_1.TIF]
