# Supplementary material for: Genomic and Chemical Investigation of Bioactive Secondary Metabolites From a Marine-Derived Fungus Penicillium steckii P2648
Source: Front Microbiol. 2021 Jun 4;12:600991. doi: 10.3389/fmicb.2021.600991 (PMC8211754; doi:10.3389/fmicb.2021.600991)
Supplement: Supplementary Table S2 — Primers used in this study. [file Table_2.docx]

**Table S2.** Primers used in this study

| Name | Sequence (5^’^-3^’^) |
| --- | --- |
| ITS1 | TCCGTAGGTGAACCTGCGG |
| ITS4 | TCCTCCGCTTATTGATATGC |
| CitsF1 | CTTGAACACCCAGCAACTCT |
| CitsF2 | TCAGCGACAGTTGACGATAC |
| CitsR1 | CATTGGCTCGGACTATCTTT |
| CitsR2 | ACTTGGAGACCGCAATAGAA |
| CitshphF | aaattccgtcaccagccctgggttgTGTCACGATTTTACACTGCA |
| CitshphR | GCTCCTTCAATATCAGTTAACGTCGGATTGATAATCGCAGTAAGA |
| CitsRTR | ATGAGTGATAGAGCCGTTGC |
| CitsRTF | GCTGTCTGTCCTGGGATGTT |
| hphsF | cgacgttaactgatattgaa |
| hphsR | CAACCCAGGGCTGGTGACGG |
| ActinRTF | CTGACCGACTACCTGATGAA |
| ActinRTR | GACGTTAGGCTGGAACAGAG |
